# Assessments of Ceanothanes Triterpenes as Cholinesterase Inhibitors: An Investigation of Potential Agents with Novel Inspiration for Drug Treatment of Neurodegenerative Diseases

**DOI:** 10.3390/metabo12070668

**Published:** 2022-07-20

**Authors:** Evelyn Muñoz-Nuñez, Soledad Quiroz-Carreño, Edgar Pastene-Navarrete, David S. Seigler, Carlos Céspedes-Acuña, Ignacio Martínez Valenzuela, Martina Oppliger Muñoz, Alexis Salas-Burgos, Julio Alarcón-Enos

**Affiliations:** 1 Grupo de Investigación Química y Biotecnología de Productos Naturales Bioactivos, Laboratorio de Síntesis y Biotransformación de Productos Naturales, Departamento de Ciencias Básicas, Facultad de Ciencias, Universidad del Bío-Bío, Chillán 3800708, Chile; evdmunoz@gmail.com (E.M.-N.); sole.m.quiroz.c@gmail.com (S.Q.-C.); edgar.pastene@gmail.com (E.P.-N.); cespedes.leonardo@gmail.com (C.C.-A.); 2 Department of Plant Biology, Herbarium, University of Illinois at Urbana-Champaign, Champaign, IL 61801, USA; seigler@life.illinois.edu; 3 Department of Pharmacology, Faculty of Biological Sciences, Universidad de Concepción, Concepción 4030000, Chile; ignmartinez@udec.cl (I.M.V.); moppliger@udec.cl (M.O.M.); alsalas@udec.cl (A.S.-B.)

**Keywords:** acetylcholinesterase inhibitor, ceanothane triterpene, molecular docking

## Abstract

The purpose of this study was to determine the inhibitory capacity of ceanothanes triterpenes isolate from Chilean Rhamnaceae on acetylcholinesterase (AChE) and butyrylcholinesterase (BChE) enzymes. Seven ceanothanes triterpenes were isolated from aerial parts of plant material by classical phytochemical methods or prepared by the hemisynthetic method. Structures were determined by the spectroscopic method (^1^H-NMR and ^13^C NMR) and mass spectrometry (MS). AChE and BChE activity were determined by the Ellmann method for all compounds. All tested compounds exerted a greater affinity to AChE than to BChE, where compound **3** has an IC_50_ of 0.126 uM for AChE and of >500 uM to BChE. Kinetic studies indicated that its inhibition was competitive and reversible. According to the molecular coupling and displacement studies of the propidium iodide test, the inhibitory effect of compound **3** would be produced by interaction with the peripheral anionic site (PAS) of AChE. The compounds tested (**1**–**7**) showed an important inhibitory activity of AChE, binding to PAS. Therefore, inhibitors that bind to PAS would prevent the formation of the AChE-Aβ complex, constituting a new alternative in the treatment of Alzheimer’s disease (AD).

## 1. Introduction

Acetylcholinesterase (AChE) is the enzyme responsible for the degradation of the neurotransmitter acetylcholine (ACh) and is considered an important target in the development of molecules with insecticidal potential. On the other hand, this enzyme is the target of drugs for the treatment of symptoms that are produced by a decrease in ACh levels in patients affected by neurodegenerative diseases of the central nervous system (CNS), for example, AD [1].

While the physiological role of AChE in the neuronal transmission is well known, it remains the focus of pharmaceutical research, directed at treatments for Myasthenia Gravis, Glaucoma, and Alzheimer’s disease (AD). Cholinergic deficiency has been clarified to be associated with AD [2]; therefore, one of the main therapeutic strategies is to inhibit the biological activity of AChE and thus increase the level of acetylcholine in the brain. Currently, most of the drugs used to treat AD are AChE inhibitors, including the synthetic compounds tacrine, donepezil, and rivastigmine, all of which have been shown to improve the condition of AD patients to some extent [3].

**Figure 1:**
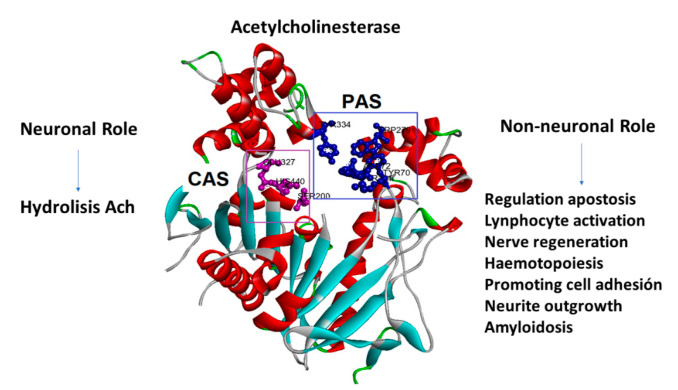
Acetylcholinesterase’s neuronal and non-neuronal roles. The amino acids that form the peripheral anionic site (PAS) are in blue, and the amino acids that form the catalytic site (CAS) are in purple.

AChE inhibitors prevent the cholinesterase enzyme from breaking down ACh, increasing both the level and duration of the neurotransmitter action. AChE has also been reported to exhibit some interesting non-cholinergic functions, including a role in cell adhesion, cell differentiation, neurogenesis, and the control of β-amyloid precursor protein processing in glial cells [4,5,6,7]. The PAS (peripheral anionic site) has been identified as the site of a number of these activities, particularly cell adhesion/neurite outgrowth and amyloidosis, located on the adjacent surface loops 37–53 and 69–96 (cell adhesion/neurite outgrowth) [8] and 275–308 (amyloidosis) [9] (Figure 1).

Cholinesterase inhibitors (also called acetylcholinesterase inhibitors) are a group of molecules that block the normal breakdown of acetylcholine. Acetylcholine is the main neurotransmitter found in the body and has functions in both the peripheral nervous system and the central nervous system. For example, acetylcholine is released by motor neurons to activate muscles; acetylcholine also plays an important role in arousal, attention, learning, memory, and motivation [10,11,12].

The main use of cholinesterase inhibitors is for the treatment of dementia in patients with Alzheimer’s disease. People with Alzheimer’s disease have reduced levels of acetylcholine in the brain. Cholinesterase inhibitors have been shown to have a modest effect on dementia symptoms such as cognition [1,13,14,15]. Cholinesterase inhibitors tend to cause side effects such as vasodilation; the constriction of the pupils in the eyes; increased secretion of sweat, saliva, and tears; slow heart rate; mucus secretion in the respiratory tract; and constriction of the airways.

The amyloid cascade hypothesis, which was first proposed in 1992, and which continues to be the leading model of AD pathogenesis, points to the deposition of amyloid-beta (Aβ) plaques in the brain as the initiating step of AD pathogenesis, which in turn leads to the accumulation of neurofibrillary tangles composed of hyperphosphorylated tau, synaptic and neuronal dysfunction and loss, and cognitive decline [15,16].

Since 1992, the discovery of a wide range of molecular and cellular processes that play a critical role in the development of AD has led experts to revise and expand the original hypothesis [17,18,19,20]. Genetic studies have also provided further insight into the complex mechanisms and biological pathways underlying AD, including those involving amyloid precursor protein (APP), tau, immune response and inflammation, lipid transport and endocytosis, synaptic function, cytoskeletal function, and axonal transport [21].

Different studies show that pentacyclic triterpenes (ursolic acid, oleanolic acid, and taraxerol), as well as some steroids (leucisterol), are capable of inhibiting AChE [22,23]. Yoo and Park in 2012 [24] demonstrate that ursolic acid inhibits the AChE competitive/non-competitive way. Lupeol and calenduladiol isolated from *Chuquiraga erinacea* showed good AChE inhibition, but greater inhibitory activity was achieved with a derivative (calenduladiol disulfate), which showed much greater inhibitory activity than its precursor [25]. The literature reviewed does not report studies on ceanothanes with AChE inhibitory activity.

According to the above, the search or synthesis of molecules with inhibitor activity on AChE is an interesting alternative to developing a new therapeutic alternative by treating the degenerative illness of the central nervous system. Many phytochemical studies are bio-directed to find biopesticides of botanical origins, which have effects on the AChE of insects. The three-dimensional structure of AChE is highly conserved evolutionarily. The folding is similar when comparing the AChE structures of *Homo sapiens* and *Drosophila melanogaster*, but there are several active site and peripheral anion site residue mutations in the *D. melanogaster* AChE structure compared to that of *H. sapiens* AChE [26]. Some taxa of the Americas, such as the Rhamnaceae family, are toxic to insects, fungi, and several bacteria strains. These effects have been associated with the presence of alkaloids, phenolics, and terpenes. Our studies of Chilean flora to develop botanical insecticides, mainly plants of family Rhamnaceae, have allowed isolated different compounds with a pentacyclic triterpenes skeleton and inhibitory activity on AChE. Some of these compounds isolated from Chilean Rhamnaceae family plants have the capacity to inhibit AChE by interaction with PAS.

## 2. Results and Discussion

**Figure 2:**
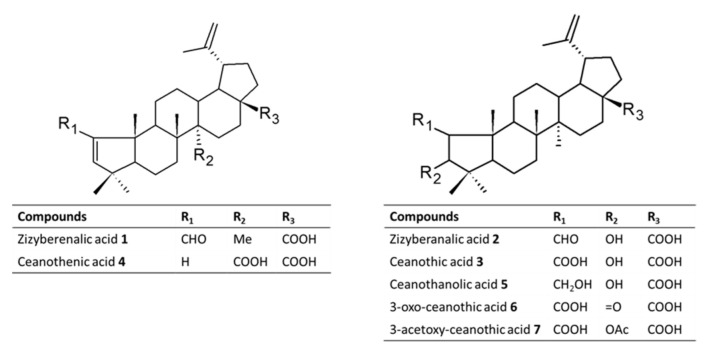
Chemical structures of Ceanothane triterpenes isolated from Chilean Rhamnaceae (compounds **1**–**5**) and derivatives prepared (**6** and **7**).

From the selected species of Rhamnaceae family growing in Chile, it was possible to isolate a set of pentacyclic triterpenes with the ceanothane skeleton (Figure 2). The literature on this family of plants indicates that these types of compounds are considered as taxonomic markers since they have not been reported in other botanical families [27]. On the other hand, the studies on the biological activity associated with this type of secondary metabolites indicate that it possesses antibacterial, cytotoxic, antiprotozoal, and insecticidal activity [27,28,29,30,31]. The analysis of the literature reveals that investigations concerning the impact of ceanothane triterpenes on the inhibition of AChE have not been undertaken so far.

### 2.1. Structural Elucidation of Ceanothane Triterpenes

Compounds **1**–**7** are pentacyclic triterpenes with ceanothane skeleton. All of them contain a 19-isopropylidene group (IR ca 1642, 881 cm^−1^; ^1^H NMR δ ca 4.71 br s; 4.89 br s; and 1.75 s Me), and a 17-carboxylic acid function (δC ca 179.0). The ^1^H and ^13^C NMR spectral data revealed their structural differences in ring A. We described below the details of their structural elucidation.

Compound **1** was obtained as a white powder. It contained an α,β-unsaturated aldehyde function. The IR spectrum displayed absorption bands at 2728, 1686 cm*^−^*^1^; ^1^H NMR δ 9.82 s, H-2; 6.49 s, H-3; ^13^C NMR δ 191.6 d, C-2; 157.0 s, C-1; 163.5 d, and C-3. These data suggested this compound to be a 1,3-didehydro derivative of ceanothic acid (compound **3**). Accordingly, compound **1** is zyziberenalic acid.

Compound **2** was obtained as a white powder. The spectral data indicated it to be closely related to compound **1**. The ^1^H NMR spectra showed signals for an aldehydic group, a vinylic methyl, five angular methyls, and two olefinic protons. The main difference was the signal at δ 9.97 ppm, attributable to the aldehyde proton, which shifted 0.31 ppm downfield with respect to the same signal in **1**, and another doublet at δ 4.16 ppm, which was assumed to correspond to the signal for the proton germinal to the 3β-hydroxyl group. The ^13^C NMR showed one oxygenated methine (δ 81.08 ppm). Consequently, several 2D-NMR spectra (COSY, HMQC, and HMBC) were obtained. The results served to confirm the cyclopentane nature of ring A in **2** and assigned all the signals of both carbon and proton spectra. Thus, the doublet at 9.73 ppm of the aldehyde proton showed long-range correlations with the carbon signal assigned to its vicinal methane carbon (72.3 ppm) and to the methane supporting the hydroxyl function (79.7 ppm). The signal at 4.16, corresponding to the proton germinal to the hydroxyl group, was long-range coupled to that of the aldehyde carbonyl. Additionally, the two methyl signals at 0.80 and 0.87 ppm, assigned to a gem-dimethyl moiety due to its mutual hydroxylated carbon signal at 79.7 ppm, as well as the signal for the quaternary carbon (40.4 ppm) supported both methyl groups. Finally, the splitting (dd, J = 8.5 and 4.7 Hz) of the proton germinal to the aldehyde group (2.02 ppm), along with the long-range heteronuclear correlation between the methane at 72.3 ppm and the proton signal for the angular methyl group at 0.93 ppm, confirmed the rearrangement of ring A in **2**. Accordingly, compound **2** is zyziberanalic acid, also called colubrinic acid.

Compound **3** was obtained by successive crystallization as a white powder. The IR spectrum showed absorption at 2500–3100 cm*^−^*^1^ and 1698 cm*^−^*^1^ for the carboxylic acid function. ^1^H NMR showed C-2βH δ 3.18 (s) and C-3αH, δ 4.99 (bs). Ceanothic acid revealed two carboxyls: one at δ178.7 and another at δ177.9, assignable to C-28 and C-1, respectively. The signals to δ151.3 and δ110.8 are assigned to C-20 and 29 vinyl carbons. The signal at δ 65.69 is due to C-2, and the signal at δ85.0 is due to C-3. The ^1^H NMR spectrum of zizyberanalic acid **2** showed that C-2αH is at 2.53 (dd, J = 4.7 Hz and J = 8.7 Hz), and C-3αH is at δ 4.32 (d, J = 8.7 Hz). The ^1^H NMR of ceanothic **3** showed C-2βH δ 3.18 (s) and C-3αH δ 4.99. In the literature, the ^1^H NMR of isoceanothic acid showed C-2αH δ 2.60 (d, J = 8.7 Hz) and C-3βH at δ 3.5 (m). The chemical shift and coupling constant of C-2δH of isoceanothic acid is similar to that of zizyberanalic acid and differs from that of ceanothic acid. The chemical shift of isoceanothic acid, C-3βH, is resonated at upfield (δ 3.50) compared to that of zizyberanalic acid (3βH, δ 4.32). This is due to the shielding effect of 2β-COOH in isoceanothic acid on 3βH. In zizyberanalic acid, the shielding by 2β-CHO effect on C-2αH is not prominent and hence appeared downfield (3βH, 4.32).

Compound **4** was obtained as a white amorphous powder. The IR spectrum showed absorption at 3067 cm*^−^*^1^ (CH=C), 1721 (C=O), and 1685 (C=O). ^13^C NMR indicated the presence of the 29 carbons. Carbon multiplicity, deduced from HMQC and DEPT experiments, indicated the presence of five methyl groups, nine methylene, seven methine, and seven quaternary carbons. Its spectral data were found to be similar to those of 1-norceanotha-1(3),20(30)-diene-28-oic acid, a norceanothane derivative prepared by us from ceanothic acid [32]. A cross comparison with this reference compound indicated that the signal of C-27 methyl was absent. Except for the methyl signal (δ 1.66 ppm) of the isopropenylidene substituent at C-19, this compound contained four methyl singlets, one less than the compound **3**. The ^1^H NMR displayed two coupled olefinic proton signals at δ 5.99 and δ 5.45, JAX = 5.7 Hz, assignable to H-1 and H-3, which were confirmed by an NOE experiment, which enhanced H-3 (δ 5.45, d) upon irradiation at the frequency of H-23 (δ 0.97,s) or H-24 (δ 0.89,s). Another irradiation at the signal of H-25 (δ 0.88 ppm) enhancing H-1 (δ 5.99,d) and H-24 also confirmed the assignment of H-1 and H-24.

Compound **5** is a white amorphous powder recrystallized from n-hexane. The proton decoupled ^13^C NMR spectrum shows the presence of 32 carbon atoms. A DEPT-90 subspectrum indicates five methine (CH) carbons, while DEPT-135 suggests ten methylene (CH_2_) and eight methyl groups. Nine quaternary carbons were identified in the signals that appear additionally in the proton broadband decoupled ^13^C NMR. The IR spectra showed an absorption at 3562 cm*^−^*^1^ that corresponds to an (O-H) for hydrogen bonded hydroxyl group. The signal at 1767 cm*^−^*^1^ is due to a carbonyl carbon (C=O, ester), whereas that at 1729 cm*^−^*^1^ is due to (C=O, acid).

Compound **6** was isolated as a white powder amorphous. The IR spectrum showed absorption at 3400–3250 (COOH), 2915, 2849, 1708 (C=O), 1588, 1381, and 1026 cm*^−^*^1^. ^1^H NMR is very similar to ceanothic acid **3**, being able to observe the disappearance of the signal δ 4.99 ppm; it corresponds to the H-3 product of the oxidation of the hydroxyl group in the same position. This correlates with the appearance of a signal in the ^13^C NMR at δ216.6 ppm corresponding to C=O in C-3. Compound **7** IR spectrum showed absorption at 3073 (CH=C), 1731 (ester), and 1681 (carbonyl) cm*^−^*^1^. Carbon multiplicity, deduced from HMQC (heteronuclear multiple quantum coherence) and DEPT (distortionless enhancement by polarization transfer) experiment, indicated the presence of seven methyl groups, nine methylene groups, seven methine groups, and nine quaternary carbons. The ^1^H NMR spectrum of **7** displayed seven three-proton singlets at δ 0.88, 0.98, 0.99, 1.06, 1.16, and 2.03 ppm, consistent with the methyl groups attached to the quaternary carbons. The presence of the acetyl groups was confirmed by the HMBC (heteronuclear multiple bond coherence) correlation of the methyl at δ 2.03 ppm with carbonyl, whereas an isopropenyl group was assigned from NMR signals corresponding to a methyl to a methyl (δH 1.68 ppm attached to a sp2-carbon (δC 152.0 ppm) showing HMBC correlation with two vinylic protons at δ 4.58 ppm and δ4.70 ppm.

### 2.2. Enzyme Inhibition and Kinetics Assays

**Figure 3:**
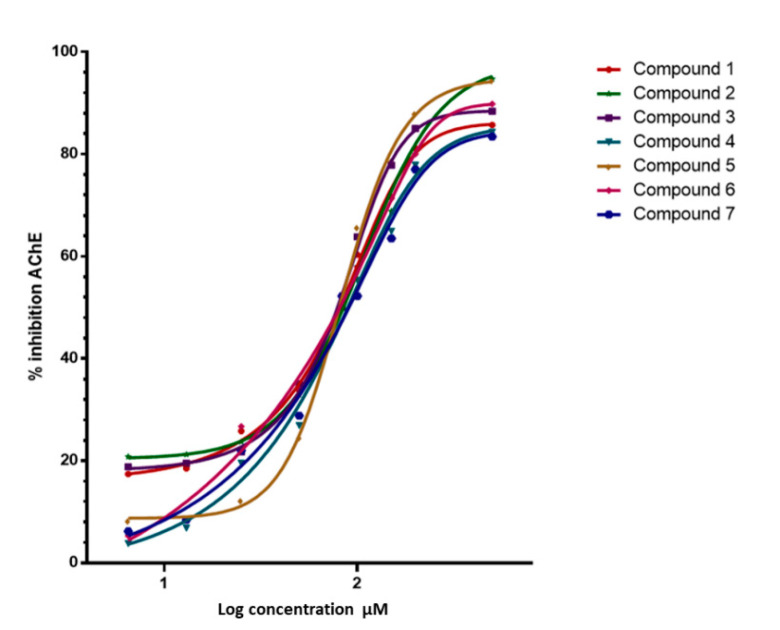
Inhibitory activitie of Ceanothanes **1**–**7** on AChE from Electrophorus electricus. Dose-dependent inhibitory activities of the seven compounds against AChE. The initial compound concentration was 50 μM and was subsequently diluted to get the set concentration of 0.0032, 0.016, 0.08, 0.40, 2.00, 10.00, and 50.00 μM. Galantamine was used as a positive control for AChE inhibition.

The inhibitory capacity of these triterpenes on AChE and BuChE was studied using a colorimetric method as described in the methodology [33] and galantamine hydrobromide as the reference compound. The results of these assays are summarized in Figure 3 and Table 1. The compounds showed high affinity for AChE IC_50_ = 0.125 µM for the commercial enzyme and 0.146 µM for the enzyme extracted from human blood and very low affinity for BuChE (IC_50_ > 500 µM). In addition, as shown in Table 1, the results obtained when evaluating the compounds with commercial AChE or with the enzyme extracted from human blood do not differ significantly.

**Figure 4:**
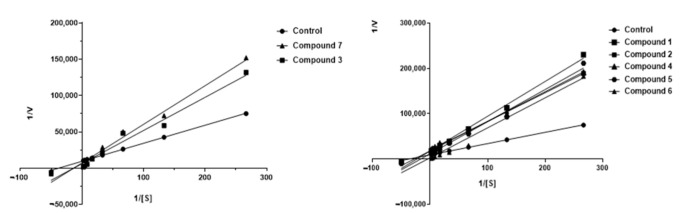
Kinetic study on the mechanism of AChE inhibition by ceanothane **2** (■), **3** (■), **6** (▲), **7** (▲), and control (●). Overlaid Lineweaver–Burk reciprocal plots of AChE initial velocity at increasing substrate concentration (0.015–0.50 µM) in the absence of inhibitor and in the presences of different concentrations of ceanothane **2** (■), **3** (■), **6** (▲), and **7** (▲). [AChE] = 0.25 U/mL.

Graphical analysis of the Lineweaver–Burk plot gives information about the binding mode. As shown in Figure 4, the lines cross the first quadrant at the same point, and Vmax decreases as the concentration of compound **3** and **7** increases. The Lineweaver–Burk plot reveals that **3** and **7** are partially competitive AChE inhibitors.

**Figure 5:**
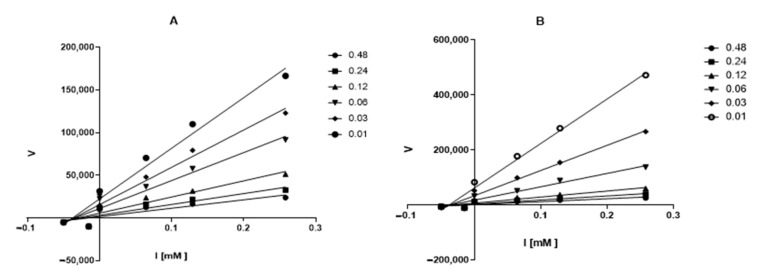
Dixon’s plot obtained for AChE in presence of increasing concentrations of ceanothanes **3** (**A**) and **7** (**B**). The initial compound concentration of compounds **3** (**A**) and **7** (**B**) was 0.48 μM and was subsequently diluted to get the set concentration of 0.01, 0.03, 0.06, 0.12 and 0.24 μM. [AChE] = 0.25 U/mL.

With the Dixon graphs (Figure 5), it is clear that the type of inhibition for ceanothane **3** and **7** is competitive since they intersect in the second quadrant, and an increase in the concentration of substrate (inhibitor) generates a line parallel to the X-axis, which indicates that the rate does not change, even when the inhibitor concentration changes. The other ceanothanes (**1**, **2**, **4**, **5**, and **6**) depict profiles that were fitted to a mixed competitive mode inhibition (Figure 3).

The above was inferred from the calculation of alpha, whose value is greater than one, this means that these compounds can act directly on the enzyme or the substrate enzyme complex.

### 2.3. Docking Studies

The docking binding energy for the compounds (**1**–**7**) had a good correlation with the experimental IC_50_ values (0.8831 Pearson products at the 95% confidence level) (Table 1). The compounds that show most negative downlink energy (**2**, **3**, and **5**) was bound to the active site by two or three conventional hydrogen bonds with residues near to aromatic cavity such as Ser 286, Phe 288, Arg 289, and Phe 330, and π-sigma, π-alkyl, and alkyl interactions with other residues of PAS (e.g., Tyr 70, Tyr 121, Trp 279, Phe 331, and Tyr 334). In contrast, compounds with higher binding energy and IC_50_ value present none or just one hydrogen bond; such is the case of compound **4**. In the case of compound **7**, an unfavorable negative–negative interaction with the residue Aps 285 was observed. It is a plausible explanation for its lower affinity.

**Figure 6:**
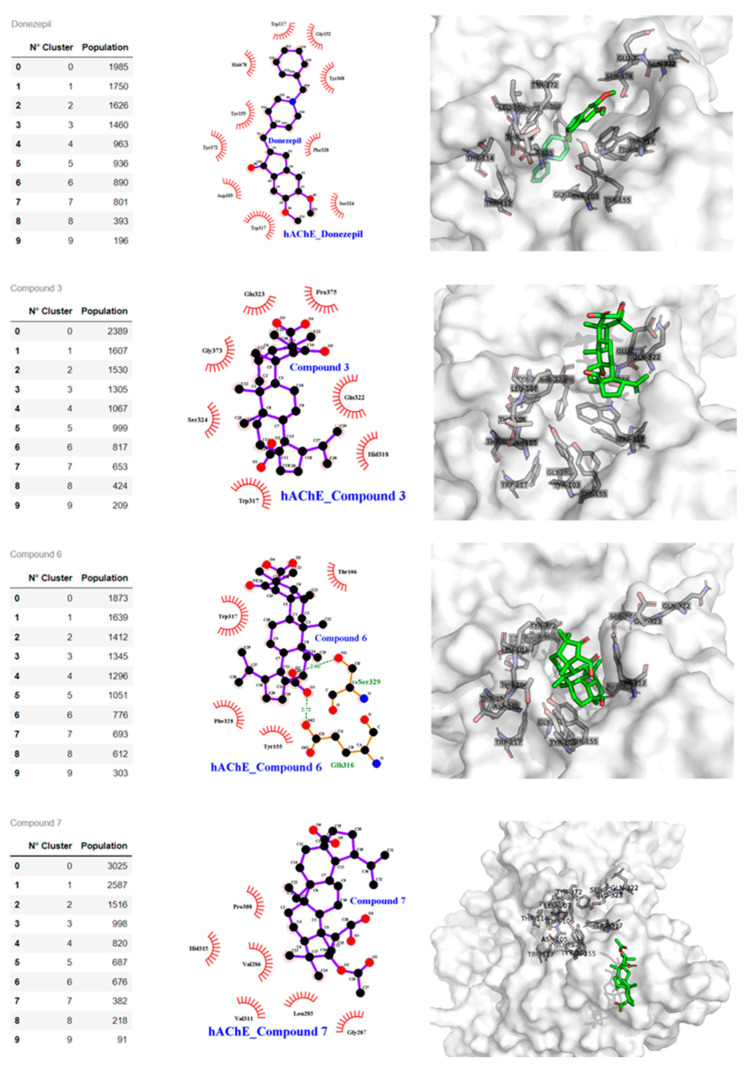
Compound binding cluster analysis from MDS and atomic interactions details. Left panel. Clustering from MDS of the AchE structures and frames population. The centroid of the most populated frame is analyzed with Ligplot and Pymol software. Central panel shows the compound and human AChE interaction at atomic level for Donepezil, compounds **3**, **6**, and **7**. Right panel: 3-D compounds and human AchE interaction; the PAS pocket residues are shown with sticks and labels, the surface is white and transparent, the compounds are with green sticks, and oxygen atoms are in red.

**Figure 7:**
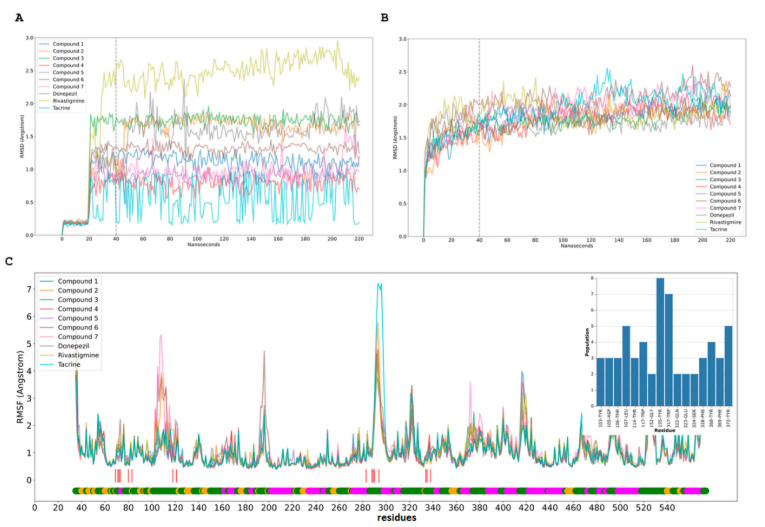
Molecular dynamics simulation (MDS) of compound and human AchE. (**A**,**B**). RMSD of compounds (**A**) and human AChEs (**B**) from MDS trajectory. RMSD for each compound assay with the line after 20 ns of equilibration with restraints; 20 ns after release the restrictions, the last 180 ns were used to RMSF calculations. (**C**). Human AChEs RMSF from MDS trajectory. These RMSF plots contain the low bar with the secondary structure f AChE, coil (green), sheet (yellow), and helix (magenta); the red line shows the more populated residues from the cluster analysis for each MDS and the compounds’ interaction.

The complex obtained for compound **3** was evaluated by molecular dynamics (MD) simulation. In the last 3 ns of simulation, it was observed that this compound interacts with residues of aromatic cavity Phe 288 and Phe 331 by a stronger conventional hydrogen bond of 2.10 ± 0.28 Å and 2.13 ± 0.28 Å, respectively, and a less strong unconventional hydrogen bond with Ile 287 2.74 ± 0.26 Å. These bonds play a crucial role in the inhibitory process. Another additional interaction is between Trp 279 and ring B, D and a methyl group by a triple perpendicular pi-alkyl interaction (Figure 6). The role of water molecules that stabilize the complex by forming hydrogen bonds into the gorge cavity and in the external zone of the active site, mostly with carboxylates anions, is also important. Furthermore, this functional group works like an intermediator for long electrostatic contacts with other residues such as Trp 279:O39 in the external zone, and Phe 330:O24 in the opposite site, Figure 7. Another factor that influences the inhibitor action is the steric effects of the voluminous structure of compound **3** in the gorge, staying in the PAS by the effect of constriction over Phe330-Tyr 121, see Figure 4. In the relaxation time, the root mean square deviation (RMSD) was smaller than 1.5 Å.

### 2.4. Propidium Iodide Displacement Assay

The PAS-AChE binding capability of ceanothanes **1**–**7** was evaluated by propidium iodide displacement assay at the concentration 0.75 and 1.5 mM. Propidium iodide is known for ability to specifically binding to the PAS region of AChE. This assay is based on the change in fluorescence that undergoes a test solution as a result of competition between propidium iodide and the compounds to be tested.

All ceanothanes were examined for their ability to bind to the PAS of EeAChE and competitively displace propidium iodide. The results are presented in Table 2. The compounds decreased in fluorescence intensity by 24–34% at 0.75 µM and by 40–44% at 1.5 µM. The results of the assay indicate that the ceanothanes assayed are capable of interacting specifically with the PAS region of AChE, corroborating the information provided by the docking approach. Based on the molecular docking study of the most active compound **3**, it was possibly inferred that this active compound displayed a significant binding interaction with the PAS. This biological profile highlights the importance of these molecules as a prototype for the development of new protective and regenerative drugs for the potential treatment of neurodegenerative diseases. The above mentioned in the text is based on the information available from the studies about the mechanisms of inhibition of AChE that indicated that there are two important sites where the inhibitors of this enzyme, such as the PAS and the catalytic site (CAS), are joined; there is even the possibility that some do so in both [11,33]. Studies in vitro have suggested that AChE may interact with beta-amyloid to promote the deposition of amyloid plaques in the brain of patients with AD [29]. This action of AChE is primarily mediated by the PAS, through which it co-localizes with the Aβ peptide and promotes Aβ fibrillogenesis by forming a stable AChE-Aβ complex [7,34]. The binding of ligands to PAS could limit the catalytic efficiency of AChE via steric and electrostatic blockage of the inhibitors’ trafficking, generating conformational changes in the active site [35].

The evidence suggests that the PAS, besides its role in the allosteric regulation of AChE-catalysed hydrolysis, also mediates heterologous protein associations that contribute to cell recognition and adhesion processes during synaptogenesis, and the nucleation of amyloid peptides during the onset of AD in humans and mammalian model systems.

The ligands or bonding by PAS and the subsequent penetration of the AChE-gorge are essential, implying the role of both the peripheral anionic site and the formation of cation–π interactions in the ligand entrance. In particular, the simulation with our molecules shows the important role of this residue in anchoring the ligand at the PAS of the enzyme and in its positioning before the gorge entrance. Once the ligand is properly oriented, the formation of specific and synchronized cation–π interactions with our molecules enables the gorge penetration. Eventually, the ligand is stabilized in a free energy basin through cation–π interactions with ceanothanes.

The inhibitory effect on AChE of terpene-related compounds has been previously reported. For instance, from Buxus baleraica Wild Sauvaitre et al., the tetracyclic triterpene N-3-isobutyrylcycloxobuxidine-F was isolated [36]. This compound was able to inhibit both CAS and PAS of AChE. In early works such as Eubanks et al. [37], using computational modeling of the THC-AChE interaction, it was discovered that THC from Cannabis also enters the binding pocket of AChE PAS. Several terpenes have affinity by the hydrophobic pocket of AChE [38]. However, to date, there are no reports regarding the proposed mechanism of AChE inhibition by ceanothanes triterpenes. It is known that the Rhamnaceae family is a rich source of pentacyclic triterpenes, particularly with the ceanothane skeleton [28,39]. These inhibitors had low micromolar IC_50_ (0.126–0.188 µM) values for AChE (Table 1). Alkaloids that were isolated from the active extracts of Esenbeckia leiocarpa (Rutaceae), leptomerine and kokusaginine, with IC_50_ values of 2.5 and 46 µM, respectively, were observed to elicit AChE inhibitory activity [40] or galangin with an IC_50_ of 120 µM [41], showing that our values are in these ranges. The kinetic analysis demonstrated that the compounds tested exhibited a competitive-type of inhibition on AChE. It is noteworthy that the Ki of our compounds is in the same range as galantamine.

Pentacyclic triterpenoids generally exhibit low oral bioavailability; in particular, compounds with the oleanane, ursane, or ceanothane skeleton fall into class IV, according to the Biopharmaceutical Classification System, due to low aqueous solubility and poor intestinal permeability. Our studies in silico about the bioavailability of the compounds assayed using SwissASDME software show good gastrointestinal absorption but a low capacity for the cross of barrier hematoencephalic (BBB), a significant characteristic or requirement of compounds is that they act in SNC. Penetrating BBB may be achieved by modifying compounds’ liposolubility by transforming the functional groups present in the molecules assayed [42,43,44].

## 3. Materials and Methods

### 3.1. Equipment and General Experimental Procedures

NMR spectra were recorded on a Bruker spectrometer 400 MHz (Palo Alto, CA, USA) using CDCl_3_ as the solvent and TMS as the internal standard. Chemical shifts are reported in δ units (ppm) and coupling constants (*J*) in Hz. Optical rotations were carried out on an ATAGO POLAX-2 L semiautomatic polarimeter. IR spectra were recorded on a Shimadzu FTIR-8400 infrared spectrophotometer. Silica gel (Kieselgel-mesh 0.15/0.30, Merck, Darmstadt, Germany) was used for all liquid chromatography procedures (LC). For thin layer chromatography (TLC), silica gel GF_254_ was used as the stationary phase with a plate dimension of 20 cm × 20 cm × 0.20 mm for analytical TLC (Merck, Darmstadt, Germany) and 20 cm × 20 cm × 0.25 mm for semi-preparative TLC (SPTLC) (Merck, Darmstadt, Germany). Spots on chromatogram were visualized under UV light and by spraying with 5% H_2_SO_4_ in methanol, and then heating at 110 °C for 5 min. Melting points were measured with a Kofler hot-stage apparatus and are reported uncorrected. Acetylcholinesterase (from *Electrophorus electricus*), 5,50-dithiobis-(2-nitrobenzoic acid) (DTNB), acetylthiocholine iodide, and butyrylcholinesterase (from equine serum) were purchased from Sigma-Aldrich.

### 3.2. Plant Materials

The aerial parts and roots of *Talguenea quinquenervia* (Gill.et Hook) Johnston were collected on the roadside at a pass 4.7 km NW of Portezuelo on the road to Ninhue (36°34.1′05″ S, 72°26.8′65″ W), VIII Region, Chile in June 2014. Voucher specimens have been deposited in the Herbarium of the Basic Science Department, University of Bio-Bio (Voucher DS-2010/05-16246) and the Herbarium of the University of Illinois at Urbana-Champaign, IL, USA (ILL, Voucher DS-16246). The aerial part of *Trevoa trinervis* Miers was collected in the San Antonio city, V Región, Chile in the summer of 2015. Voucher specimens were deposited in the Herbarium of the Basic Science Department, University of Bio–Bio. *Colletia spinossisima* Gmelin was collected in Colbun Lake, VII Region, Chile in the summer of 2015. Voucher specimens were deposited in the Herbarium of the Basic Science Department, University of Bio–Bio Bio and the Herbarium of the University of Illinois, at Urbana-Champaign, IL, USA, (ILL, Voucher DS-16252). *Discaria chacaye* (G. Dom.) Tortosa was collected on the road to Yungay (37°06′57″ S, 72°15′25″ N), VIII Region, Chile. Voucher specimens have been deposited in the Herbarium of the Basic Science Department, University of Bio–Bio and Herbarium of the University of Illinois, at Urbana-Champaign, IL, USA (ILL, Voucher DS-16253).

### 3.3. Extraction and Isolation

The leaves and stems (1.5 kg) of each plant under study separality were milled and fourfold extracted for 48 h with MeOH, at room temperature, and the combined macerate was filtered and evaporated under reduced pressure. The crude extract (180 g) was partitioned by being dissolved in a mixture of MeOH/H_2_O (1:2), transferred to a separating funnel, and extracted with n-hexane (15 × 300 mL) and with ethyl acetate (EtOAc) (10 × 250 mL). Both n-hexane and EtOAc fractions were concentrated under reduced pressure, and the aqueous phase was concentrated by lyophilization.

**Figure 8:**
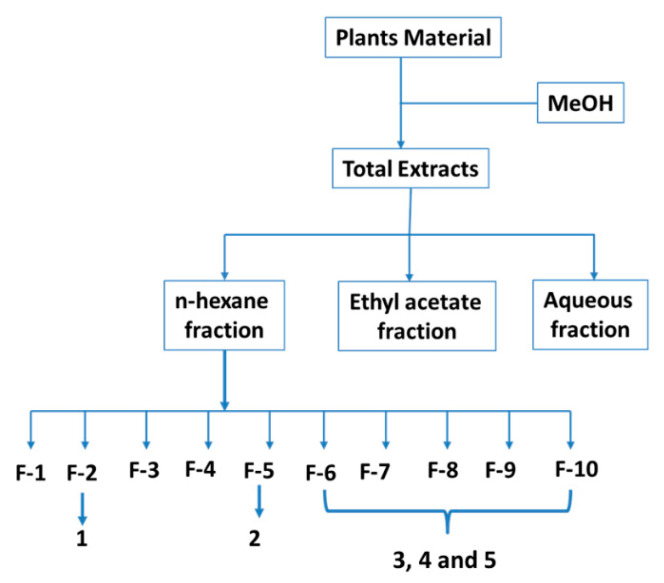
Method of obtaining extracts, fractions, and compounds.

The *n*-hexane fraction was subjected to LC (Silica Gel 60, 63–200 μm) starting with *n*-hexane (100%), gradually enriching with EtOAc (0 to 100%) to give 70 column fractions. Column fractions were monitored by thin-layer chromatography (TLC) (Silica Gel 60 F254). Fractions with similar or identical Rf (TLC patterns) were collected to provide 10 major fractions (F1, F2, F3, F4, F5, F6, F7, F8, F9, and F10). Among them, fraction F2 was subjected to quick column chromatography using n-hexane: EtOAc (90:10) as the eluting solvent to give compound **1** (50 mg) after crystallization from acetone. Compound **2** (120 mg, after crystallization from acetone) was obtained from fraction F5 by flash columnchromatography using n-hexane: EtOAc (70:30) as the eluting solvent. Fractions F6 to F10 were combined and purified by repeated preparative TLC, and after recrystallization from MeOH, afforded compound **3** (300 mg). Additionally, compound **3** was also obtained from the n-hexane extracts of *C. spinossisima* and *D. chacaye*. A similar process was conducted with n-hexane extract from *T. trinervis* to afford **4** (150 mg) and **5** (130 mg) (Figure 8).

### 3.4. Spectroscopic Data

Zizyberenalic acid (**1**): Mp 215–216°[lit mp 218–220 °C] (Kundu et al. 1989); [α]_D_^20^ + 24 (c = 0.50, MeOH); IR (KBr) υ_max_ cm^−1^: 2500–3100 (COOH), 2728 (CHO), 1714 and 1686 (C=O), and 1642 and 881 (C=CH_2_). ^1^H NMR (CDCl_3_): δ 9.66 (s). 6.52 (s), 1.56 (m), 1.50 (m), 1.54 (m), 1.44 (m), 1.67 (m), 2.05 (m), 1.64 (m), 1.73 (m), 1.09 (m) 2.18 (m), 2.01 (m), 1.43 (m), 1.61 (m), 1.18 (m), 1.91 (m), 2.99 (bs), 2.30 (m), 1.44(m), 1.99 (m), 1.48 (m), 0.95 (s), 0.94 (s), 0.96 (s), 1.10 (s), 1.11 (s), 4.71 (s), 4.58 (s), and 1.65 (s). ^13^C NMR (CDCl_3_): δ 191.45 (C-2), 157.37(C-1), 163.36(C-3), 43.81(C-4), 63.09(C-5), 16.86(C-6), 35.13(C-7), 42.59 (C-8), 49.45(C-9), 52.22(C-10), 24.14(C-11), 25.16(C-12), 38.26(C-13), 42.99(C-14), 30.59(C-15), 29.84(C-16), 56.17(C-17), 47.55(C-18), 46.97(C-19), 150.07(C-20), 32.36 (C-21), 37.14(C-22), 28.19(C-23), 16.86 (C-24), 19.07(C-25), 17.67(C-26), 14.76(C-27), 180.88(C-28), 109.68(C-30), and 19.31(C-29).

Zizyberanalic acid (**2**): Mp 264–266°[ lit mp 263–265 °C](Kundu et al., 1989), [α]_D_^24^ + 3°; IR (KBr) υ_max_ cm^−1^: 3380 (OH), 1717, 1698 (COOH/CHO), 1644, and 828 (=CH_2_). ^1^H NMR(CDCl_3_): δ 9.97 (s, H-2), 2.53 (dd, J = 4.4; 8.8 Hz, H-1),4.32 (d, H-3), 1.54 (m, H-5), 1.52 (m, H-6), 1.49 (m, H-7), 1.41 (m, H-7), 1.65 (m, H-9), 2.04 (m, H-11), 1.64 (m, H-11), 1.81 (m, H-12), 1.25 (m, H-12), 2.64 (m, H-13), 2.02 (m, H-15), 1.40 (m, H-15), 1.60 (m, H-16), 1.19 (m, H-16), 1.71 (t, H-18), 3.43 (bs, H-19), 2.31 (m, H-21), 1.44 (m, H-21), 1.98 (m, H-22), 1.47 (m, H-22), 0.97 (s, H-23), 0.93 (s, H-24), 1.00 (s, H-25), 1.01 (s, H-26), 1.03 (s, H-27), 4.76 (s, H-30), 4.64 (s, H-30), and 1.71 (s, H-29). ^13^C NMR (CDCl_3_): δ 206.1(C-2), 73.9(C-1), 80.9(C-3), 41.2(C-4), 63.0(C-5), 18.5 (C-6), 34.6(C-7), 42.3(C-8), 50.5(C-9), 48.2(C-10), 24.9 (C-11), 25.6(C-12), 38.5(C-13), 43.1(C-14), 30.4(C-15), 32.9(C-16), 56.5(C-17), 49.7(C-18), 47.8(C-19), 151.2(C-20), 31.2(C-21), 37.6(C-22), 26.3(C-23), 25.6(C-24), 14.8(C-25), 17.3(C-26), 15.0(C-27), 178.8(C-28), 109.96(C-30), and 19.4(C-29).

Ceanothic acid (**3**): Mp. 332–334 °C [lit mp 328–331 °C] (Kundu et al. 1989); [α]_D_^24^ + 38° (c = 0.8, MeOH); IR (KBr) υ_max_ cm^−1^: 2500-3500 (m, COOH, OH), 1690 (C=O), and 1640 and 890 (C=CH_2_). ^1^H NMR (pyridine-d_5_): δ.18(s, H-1), 4.99 (s, H-3), 1.55 (m, H-5), 1.45 (m, H-6), 1.84 (m, H-7), 1.76 (m, H-7), 1.60 (m, H-9), 2.05 (m, H-11), 1.34 (m, H-12), 2.94 (m, H-13), 1.92 (m, H-15), 2.61 (d, H-16), 1.50 (d, H-16), 1.79 (m, H-18), 3.93 (bs, H-19), 2.23 (m, H-21), 1.50 (m, H-21), 2.23 (m, H-22), 1.50 (m, H-22), 0.81 (s, H-23), 0.92 (s,H-24), 0.88 (s, H-25), 0.99 (s, H-26), 1.00 (s, H-27), 4.7 (s, H-30), 4.60 (s, H-30), and 1.66 (s, H-29). 13C NMR (pyridine-d5): δ 177.9 (C-2), 67.2 (C-1), 85.0 (C-3), 43.9 (C-4), 57.1 (C-5), 19.2 (C-6), 34.9(C-7), 43.7 (C-8), 45.2 (C-9), 49.7 (C-10), 24.4 (C-11), 26.4 (C-12), 39.3 (C-13), 42.3 (C-14), 30.7 (C-15), 33.1 (C-16), 56.8 (C-17), 50.1 (C-18), 47.7 (C-19), 151.3 (C-20), 31.5 (C-21), 37.6 (C-22), 31.6 (C-23), 20.1 (C-24), 19.05 (C-25), 17.1 (C-26), 15.2 (C-27), 178.7 (C-28), 110.8 (C-30), and 19.7 (C-29).

Ceanothenic acid (**4**): Mp 350–354 °C [lit mp >300 °C], [α]D24 −15°. IR νmax cm^−1^ 2500-3000 (COOH, OH), 1683 (C=O), and 1641 and 881 (C=CH_2_). ^1^H NMR (pyridine-d5): δ 5.55 (1H, d, *J* = 5.7 Hz, H-1), 5.01 (1H, d, *J* = 5.0 Hz, H-3), 0.86 (1H, m, H-5), 1.07 (2H, m), 1.25 (1H, m, H-7α), 1.33 (1H,m, H-7β), 1.48 (1H, dd, *J* = 3.2;12.3 Hz, H-9), 1.20 (2H, m), 1.30 (1H, m, H-12α), 1.74 (1H, m, H-12β), 2.02 (1H, m, H-13), 1.00 (1H, m, H-15), 0.95 (1H, m, H-16α), 1.99 (1H, m, H-16β), 1.36 (1H, m, H-18), 2.72(1H, dt, *J* = 7.5;4.0 Hz, H-19), 1.00 (1H, m, H-21α), 1.57 (1H, m, H-21β), 1.00 (1H, m, H-22α), 1.56 (1H, m, H-22β), 0.59 (3H, s, H-23), 0.53 (3H,s, H-24), 0.60 (3H, s, H-25), 0.68 (3H, s, H-26), 1.32 (3H, s, H-29). 4.22 (1H, d, *J* = 2.2 Hz, H-30a), 4.35 (1H, d, *J* = 2.1 Hz, H-30b). ^13^C NMR (pyridine-d5): δ140.1 (C-1), 138.2 (C-3), 44.0 (C-4), 61.9 (C-5), 16.88 (C-6), 36.9 (C-7), 40.7 (C-8), 47.4 (C-9), 50.0 (C-10), 22.3 (C-11), 25.2 (C-12), 39.0 (C-13), 59.3 (C-14), 27.4 (C-15), 33.7 (C-16), 55.6 (C-17), 50.9 (C-18), 46.6(C-19), 149.7(C-20), 29.8 (C-21), 36.5 (C-22), 28.6(C-23), 20.5 (C-24), 19.3(C-25), 17.2 (C-26), 178.5 (C-27), 177.8 (C-28), 17.9 (C-29), and 109.1 (C-30).

Ceanothanolic acid (**5**): Mp 286–288 °C [lit mp 286 °C](Lee et al., 1997). [α]D_18_ ^15^° (c = 0.50, pyridine). IR υmax cm^−1^: 2500-3500 (COOH, OH), 1683 (C=O), and 1641 and 881 (C=CH_2_). ^1^H NMR (pyridine-d5): δ 4.36 (dd, *J* = 4.6, 10 Hz, H-1), 4.06 (dd, *J* = 8.6, 10 Hz, H-1), 1.94 (dt, *J* = 4.6, 8.6 Hz, H-2), 4.15 (d, *J* = 8.6 Hz, H-3), 2.68 (dt, *J* = 3.5 Hz, 12.1 Hz, H-13), 2.60 (brd, *J* = 12.6 Hz, H-16), 1.69 (t, *J* = 11.4 Hz, H-18), 3.48 (dt, *J* = 3.9,11 Hz, H-19), 1.23 (s, H-23), 0.97 (s, H-24), 0.79 (s, H-25), 1.01 (s, H-26), 1.01 (s, H-27), 1.78 (s, H-29), 4.91 (brs, H-30), and 4.76 (brs, H-30). ^13^C NMR (pyridine-d5): δ 64.6 (C-2), 62.9 (C-1), 87.0 (C-3), 39.7 (C-4), 62.7 (C-5), 18.7 (C-6), 35.1 (C-7), 42.4 (C-8), 50.9 (C-9), 44.5 (C-10), 24.2 (C-11), 25.8 (C-12), 38.5 (C-13), 43.2 (C-14), 30.5 (C-15), 33.1 (C-16), 56.6 (C-17), 50.0 (C-18), 47.9 (C-19), 151.3 (C-20), 31.4 (C-21), 37.7 (C-22), 26.2 (C-23), 25.8 (C-24), 14.6 (C-25), 17.3 (C-26), 15.0 (C-27), 178.8 (C-28), 19.6 (C-29), and 110.0 (C-30).

3-oxo-ceanothic acid (**6**): IR (KBr) υmax cm^−1^: 3400-3250(COOH), 2915, 2849, 1708(C=O), 1588, 1381, and 1026. ^1^H NMR (CDCl_3_): 3.01 (1H, s, H-1), 2.13 (1H, ddd, *J* = 3.2, 11.8, 11.8 Hz, H-13), 2.96 (1H, ddd, *J* = 4.5, 10.8, 10.8 Hz, H-19), 4.70 (s, H-30), 4.58 (s, H-30), 2.99 (s, H-2), 2.95 (m, H-19), 1.66 (s, H-29), 1.46 (s, H-23), 1.00 (s, H-24), 0.98 (s, H-25), 0.93 (s, H-27), and 0.84 (s, H-27). ^13^C NMR (CDCl_3_): δ 170.5 (C-2), 69.2 (C-1), 216.6 (C-3), 47.2 (C-4), 59.0 (C-5), 17.3 (C-6), 33.8 (C-7), 42.8 (C-8), 45.5 (C-9), 49.6 (C-10) 23.9 (C-11), 25.1 (C12), 38.1 (C-13), 41.9 (C-14), 29.7 (C-15), 32.2 (C-16), 56.5 (C-17), 49.6 (C-18), 47.0 (C-19), 150.3 (C-20), 30.7 (C-21), 36.9 (C-22), 28.0 (C-23), 14.7 (C-24), 14.7 (C-25), 20.8 (C-26), 16.4 (C-27), 176.5 (C-28), 109.7 (C-30), and 19.4 (C-29).

3-O-acetyl-ceanothic acid (**7**): ^1^H NMR (CDCl_3_): 2.52 (1H, d, H-1), 5.07 (1H, d, H-3), 1.71 (1H, m, H-5), 1.43 (2H, m, H-6), 1.40 (1H, m, H-7), 1.97 (1H, m, H-7), 1.71 (m, H-9), 1.43 (1H, m, H-11), 1.62 (1H, m, H-11), 1.11 (1H, m, H-12), 1.62 (1H, m, H-12), 2.27 (1H, m, H-13), 1.43 (1H, m, H-15), 1.90 (1H, m, H-15), 1.43 (1H, m, H-16), 2.27 (1H, m, H-16), 1.62 (1H, m, H-18), 3.01 (1H, dt, H-19), 1.62 (2H, m, H-21), 1.43 (1H, m, H-22), 1.90 (1H,m, H-22), 1.16 (3H, s, H-23), 0.88 (3H, s, H-24), 1.06 (3H, s, H-25), 0.98 (3H, s, H-26), 0.99 (3H, s, H-27), 1.68 (3H, s, H-29). ^13^C NMR(CDCl_3_) 64.7 (C-1), 177.4 (C-2), 87.0 (C-3), 44.0 (C-4), 57.8 (C-5), 19.5 (C-6), 35.4 (C-7), 42.9 (C-8), 46.1 (C-9), 50.6 (C-10), 24.8 (C-11), 26.8 (C-12), 40.1 (C-13), 44.3 (C-14), 31.8 (C-15), 33.5 (C-16), 57.5 (C-17), 50.5 (C-18), 48.6 (C-19), 152.0 (C-20), 31.1 (C-21), 38.3 (C-22), 30.8 (C-23), 20.1 (C-24), 18.9 (C-25), 17.2 C-26), 15.3 (C-27), 180.1 (C-28), 19.7 (C-29), and 110.3 (C-30).

### 3.5. Preparation of 3-Oxo-Ceanothic Acid 6

Ceanothic acid **3** (0.05 g, 0.1 mmol) was previously dissolved in CH_2_Cl_2_ (10 mL) and then was added to a 0.04 M pyridinium chlorochromate in CH_2_Cl_2_ solution (40 mL). The resulting solution was stirred at room temperature for 2 h, and then Et_2_O (30 mL) was added. The removal of the resulting brown solid residue by filtration through a Celite pad and the evaporation of the filtrate yielded a residue that was purified on a Si gel column (120 g, 230–400 mesh) eluted with n-hexane:EtOAc (80:20) to give **6** (0.036 g, yield 72%).

### 3.6. Preparation of 3-Acetoxyceanothic Acid 7

Ceanothic acid **3** (200 mg, 0.41 mmol) was warmed with 1 mL of Ac_2_O in 0.25 mL of anhydrous-pyridine for 1 h, diluted with water, allowed to stand for 5 h, and extracted with Et_2_O. The removal of Et_2_O produced a residue that crystallized from MeOH as colorless fine needles of 7 (206 mg, yield 95%).

### 3.7. In Vitro AChE/BChE Inhibitory Activity Assay

The Ellman assay was used to test acetylcholinesterase (AChE from *Electrophorus electricus*, and human blood) and butyrylcholinesterase (BChE from equine serum) inhibition activity [33]. Human blood was drawn using sterilized syringes and stored in BD Vacutainer tubes with heparin as an anticoagulant at 4 °C for 1 h. To lyse the erythrocytes, 1 mL of a 1:50 dilution of the blood sample was prepared with the non-ionic detergent triton X-100. A mixture of the DTNB (125 μL), enzyme solution (25 μL), and compound solution (25 μL) was prepared and incubated at room temperature for 20 min. All the assays were under 0.1 M KH_2_PO_4_/K_2_HPO_4_ buffer, pH 8.0. The substrate was added to start the enzymatic reaction. The absorbance (λ = 405 nm) was recorded at a controlled temperature of 30 °C for 5 min. All measurements were performed fivefold as triplicate. The compounds were assayed in a dilution interval of 15 to 500 μg/mL. Galantamine was used as positive control. The percentage of inhibition was determined as follows:

$$I\left(\%\right)=\left(1-\frac{A_{probe}}{A_{blank}}\right)100$$

### 3.8. Kinetic Characterization of AChE Inhibition

To investigate the inhibition mechanism of the tested compounds on AChE, a kinetic analysis was performed. The experiments were carried out using a combination of four substrate concentrations and three inhibitor concentrations with the view to obtain a double reciprocal plot (Lineweaver–Burk), in which each point is the mean of three different experiments. A parallel control with no inhibitor in the mixture allowed adjusting activities to be measured at various times.

### 3.9. Propidium Displacement Assay

This trial was carried out in order to evaluate the interaction of the ceanothanes-triterpenes understudy with the PAS of AChE. A solution of the test compound or standard donezepil was incubated with five units of *Ee*AChE at 25 °C for 15 min. After incubation, 50 μL of 1 μM propidium iodide solution was added to make the final assay volume 200 μL. After 15 min, the fluorescence intensity was observed at an excitation wavelength (λex = 535 nm) and an emission wavelength (λem = 595 nm) using a fluorescence plate reader Perkin Elmer VictorX2 (Perkin Elmer, Singapur). The percentage inhibition was calculated by the following expression: 100-(IFi/IF0 × 100), where IF1 and IF0 correspond to fluorescence intensities with and without the test compound, respectively. Each assay was carried out in triplicate.

### 3.10. In Silico Assays

#### 3.10.1. Ligand Construction

All structures of the ligands were constructed using Spartan’10 1.1.0 2011, and their geometries were optimized using Density Functional B3LYP 6-31**G ab initio methods in vacuum.

#### 3.10.2. Molecular Docking

The crystallized structure of *Homo sapiens* AChE (hAChE) PDB ID: 4M0E was obtained from the protein data bank [44]. The molecular docking of hAChE and ligands 1–7 was carried out using Smina, a fork of Autodock Vina [45]. Dockings with donepezil, rivastigmine, and tacrine were used as controls. The geometries of ligands were optimized and minimized with Avogadro using the MMFF94 force field and the conjugate gradient algorithm. The grid was centered in the middle of the gorge of the catalytic site through the ligand bound to the crystallographic structure as a reference (12 Å in every direction). With regard to the macromolecule, all residues within 6 Å from the reference ligand were counted as flexible. Exhaustiveness and seed parameters had the settings 64 and 0, respectively. The RMSD threshold for multiple clustering was set to 1 Å. The result was analyzed by a ranked cluster and binding energy (ΔG), where the lower-energy and more populated cluster was selected as the best protein–ligand complex for further analyses. To test the docking accuracy, the co-crystallized ligand was re-docked under the same conditions, and an RMSD of 0.62147 Å was obtained. All experiments were made with a physiological pH.

#### 3.10.3. MD Protocol

To obtain the parameters and topology files of all compounds, the most stable docking pose of the complex was employed by using the generalized amber force field (GAFF) through the Antechamber software [46]. For the protein, it was necessary to add the missing residues with Modeller [47] before establishing the protonation state of the ionizables residues using PDB2PQR 3.3.1 software from the APBS web server [48]. The parameters derived from the ff19SB force field were used for protein [49]. The system was solvated using a water box (88.5 × 92.1 × 102.7 Å, 837,647.4 Å^3) of an optimum point charge (OPC) water model [50] using tleap from Ambertools21 [51]. Counter-ions (Na^+^) were used to maintain electroneutrality. Energy minimizations were carried out following four successive stages of minimization, wherein each minimization cycle consisted of 5000 steps using the steepest descent algorithm, followed by 5000 steps using a conjugate gradient method. In the first two-stage, a harmonic positional restraint of 500 kcal/mol xÅ^2^ was applied first over the all-protein atoms to accommodate the solvent and ions and then was applied only to heavy protein atoms to minimize the hydrogens. In the following two stages, minimizations were carried out successively, reducing the restraint from 10 to 0 kcal/mol x Å^2^, except for a restriction of 10 kcal/mol x Å^2^ over ligands. The minimized systems were equilibrated under NVT conditions, heating the system from 0 K to 310.15 K using the weak Langevin thermostat [52], within 300 ps. Then, 20 ns-long equilibrations in NPT conditions were carried out for each system, keeping the restriction of 10 kcal/mol x Å to the ligands at a constant temperature of 310.15 K using the Langevin thermostat and constant pressure of 1 atm using the Berendsen barostat [51,52]. Lastly, productions of 200 ns were realized for each system under the same conditions of the NPT equilibration without restraints. All the simulations were performed using pmemd.cuda of the Amber20 software [48], using periodic boundary conditions with a time step of 2 fs. The SHAKE algorithm for bond length constraints involving hydrogen atoms was used. Non-bonded interactions were calculated using a cut-off of 8 Å, and the Particle Mesh Ewald method [48] was used for treating long-range electrostatic interactions. An analysis was carried out with cpptraj and pytraj software [48].

## 4. Conclusions

The present work demonstrated potent AChE inhibitory and very low BuChE inhibitory activity of seven ceanothane-triterpenes isolated from Rhamnaceae plants growing in Chile. Furthermore, these ceanothanes not only bind to the AChE catalytic site but also to the PAS and in this way could inhibit the formation of senile plaques typical of Alzheimer’s disease. Currently, we are working to increase the interaction between the active site and the peripheral anionic site in the search for the enhancement of the potency of natural cholinesterase inhibitors.

## Figures and Tables

**Table 1:** Inhibition of acetylcholinesterase (AChE from Electrophorus electricus, and human blood) and butyrylcholinesterase (BChE from equine serum) from Ellman’s assays, IC_50_, Ki, Moldock value, and inhibition type for ceanothanes **1***–***7** used in this study.

Compounds		IC_50_ ± SEM, µM				Ki	MolDock
	M	AChE *	AChE hb ^a,^*	BuChE	Inhibition Type	µM	Score
**1**	452.7	0.184 ± 0.0007	0.215 ± 0.005	>500	acompetitive	0.046	−109.891
**2**	470.7	0.150 ± 0.0009	0.173 ± 0.006	>500	acompetitive	0.028	−144.641
**3**	486.7	0.125 ± 0.0004	0.146 ± 0.003	>500	competitive	0.055	−145.850
**4**	454.6	0.188 ± 0.0005	0.219 ± 0.004	>500	acompetitive	0.022	−107.504
**5**	472.7	0.155 ± 0.0001	0.181 ± 0.0007	>500	acompetitive	0.056	−141.850
**6**	484.7	0.172 ± 0.0012	0.199 ± 0.009	>500	acompetitive	0.047	−112.846
**7**	528.7	0.179 ± 0.0001	0.207 ± 0.006	>500	competitive	0.050	−121.490
Galantamine	287.4	0.046 ± 0.0005	0.040 ± 0.0004	0.739 ± 0.012	competitive	0.045	−184.840

* IC_50_ values represent the concentration of the inhibitor required to decrease enzyme activity by 50% and are the mean of the triplicate independent experiments. ^a:^ AChE was obtained from human blood samples.

**Table 2:** Inhibition of AChE and displacement of propidium iodide from the PAS.

Compound	IC_50_ µM	% Displacement of Propidium Iodide	
		0.75 µM	1.5 µM
**1**	0.184 ± 0.0007	19.7 ± 0.8	26.3 ± 1.3
**2**	0.150 ± 0.0009	17. 4 ± 0.7	23.2 ± 0.8
**3**	0.125 ± 0.0004	34.2 ± 0.6	45.6 ± 1.1
**4**	0.188 ± 0.0005	20.1 ± 1.0	26.7 ± 1.3
**5**	0.155 ± 0.0001	14.5 ± 0.3	19.3 ± 1.5
**6**	0.172 ± 0.0012	24.3 ± 0.7	40.5 ± 1.5
**7**	0.179 ± 0.0001	24. 1 ± 0.3	32.1 ± 1.3
Donepezil	0.012 ± 0.007	73.2 ± 1.9	84.6 ± 3.6

Data are mean ± SEM (n ≥ 3 experiments).

## Data Availability

Data can be requested from the corresponding author due to restrictions on privacy.
